# Supplementary material for: Impact of Short-Term (+)-JQ1 Exposure on Mouse Aorta: Unanticipated Inhibition of Smooth Muscle Contractility
Source: Cells. 2023 May 24;12(11):1461. doi: 10.3390/cells12111461 (PMC10252217; doi:10.3390/cells12111461)
Supplement: Supplementary file 1 [file cells-12-01461-s001.zip › cells-2376010-supplementary.pdf]

cells MDPI

Supplemental figures

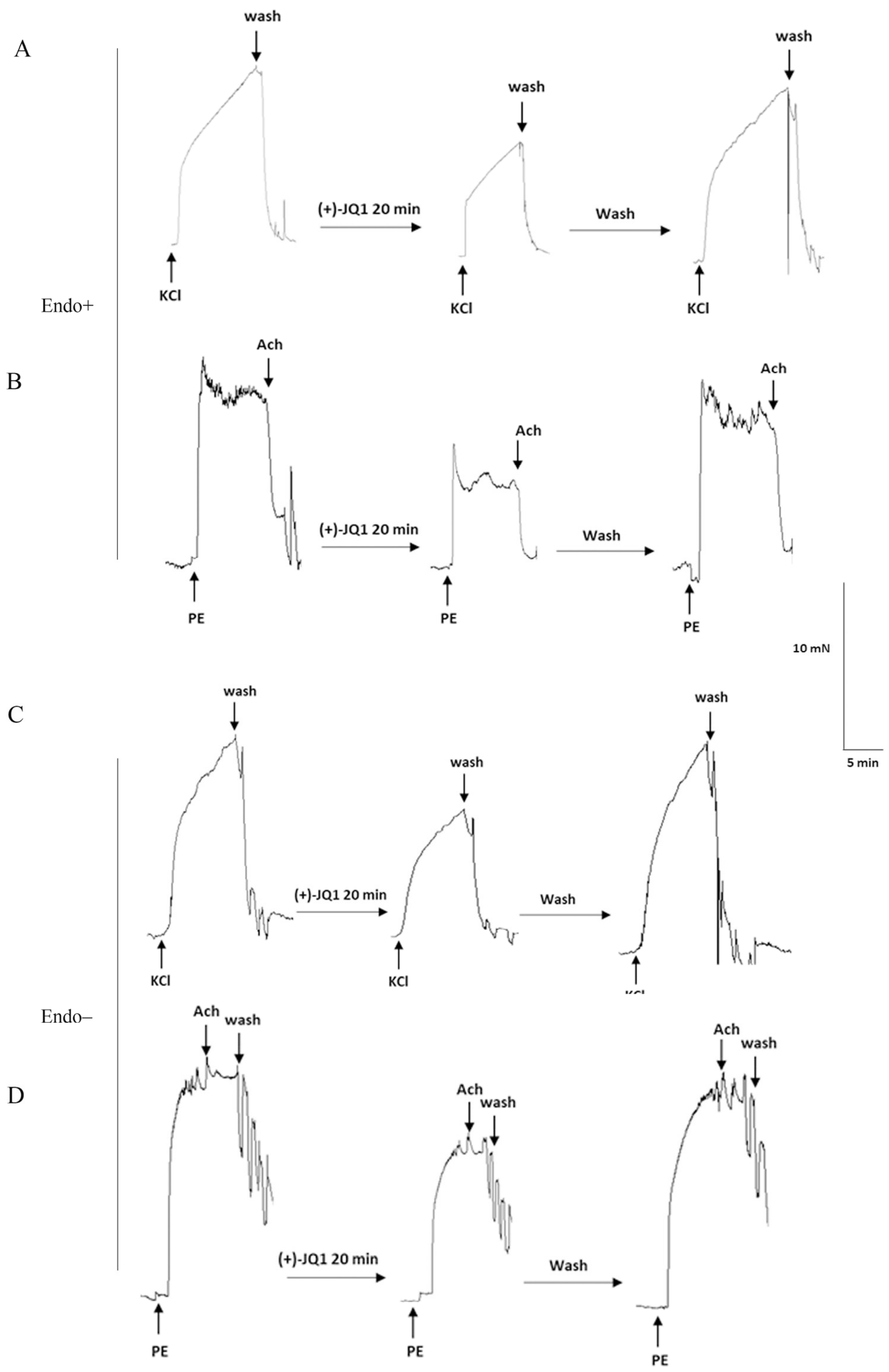

Supplemental Figure S1. The inhibitory effects of (+)-JQ1 on contractility are reversible in mouse aortas with and without intact endothelium. Representative recordings show that mouse aortas with (upper panel) or without (lower panel) an intact endothelium were pretreated with (+)-JQ1 (10 μM) for 20 min, followed by exposure to KCl (50 mM) or PE (1 μM). After washing (+)-JQ1 away from the tissue bath for 30 min, the contractile responses of KCl and PE returned to previous levels without (+)-JQ1 treatment.

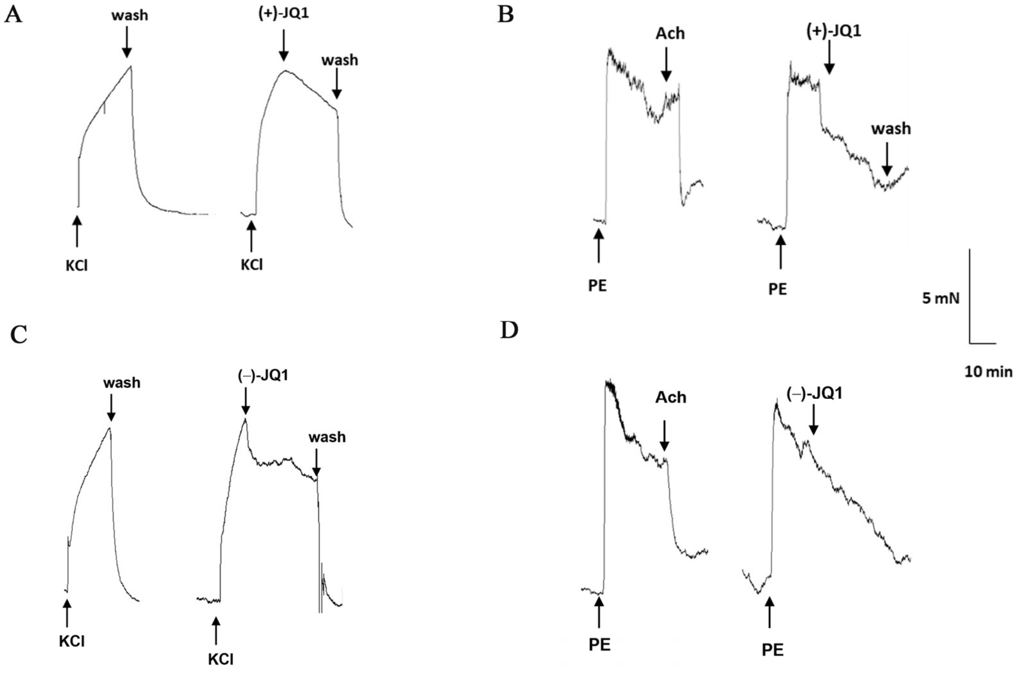

Supplemental Figure S2. (+)-JQ1 and (–)-JQ1 have vasodilating effects on mouse aortas with intact endothelium. Mouse aortas with intact endothelium were exposed to KCl (50 mM, **A** and **C**) or PE (1 μM, **B** and **D**) for 10 min. (+)-JQ1 (10 μM) or (–)-JQ1 (10 μM) was applied when the contraction reached its maximum.

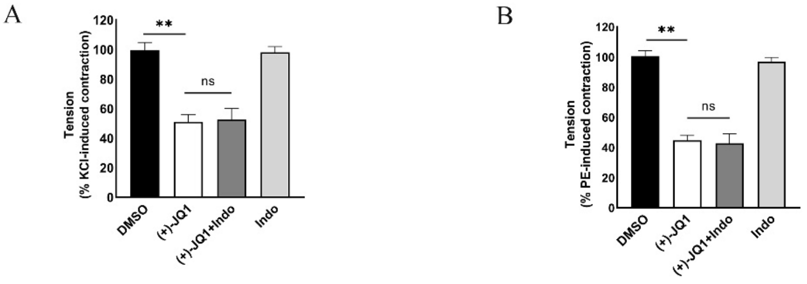

Supplemental Figure S3. Indomethacin does not affect (+)- JQ1-induced inhibitory effects on KCl and PE-induced contractility. Mouse aortic tissues with intact endothelium were pretreated without and with indomethacin (10 μM) for 20 min, followed by incubation (+)- JQ1 (10 μM) and subsequent exposure to KCl (A) or PE (B). **P<0.01, ns: not significant; n= 3.

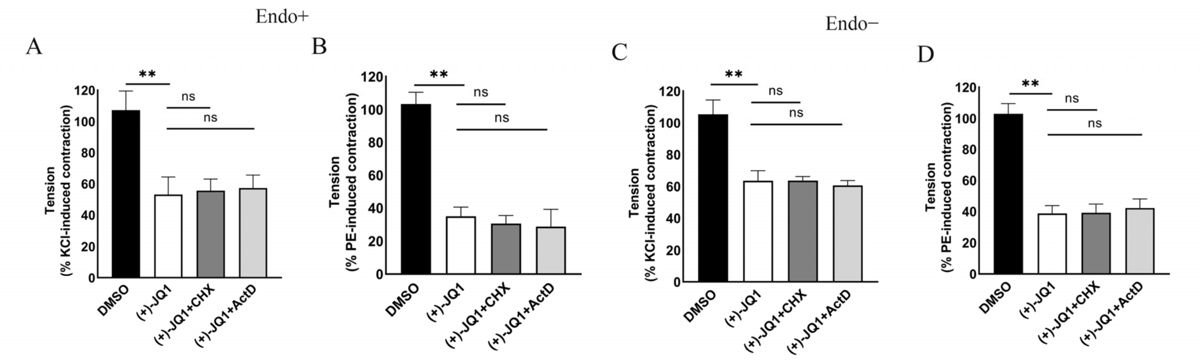

Supplemental Figure S4. The inhibitory effect of (+)-JQ1 does not involve the translational and transcriptional mechanism, no matter with endothelium or without endothelium.

Mouse aortic tissues were pretreated with CHX (10 μM) and Act D (1 μM) for 20 min, followed by incubation (+)-JQ1 (10 μM) and subsequent exposure to 50 mM KCl (A and C) or 1 μM PE (B and D).**$P<0.01$, ns: not significant; n= 4.

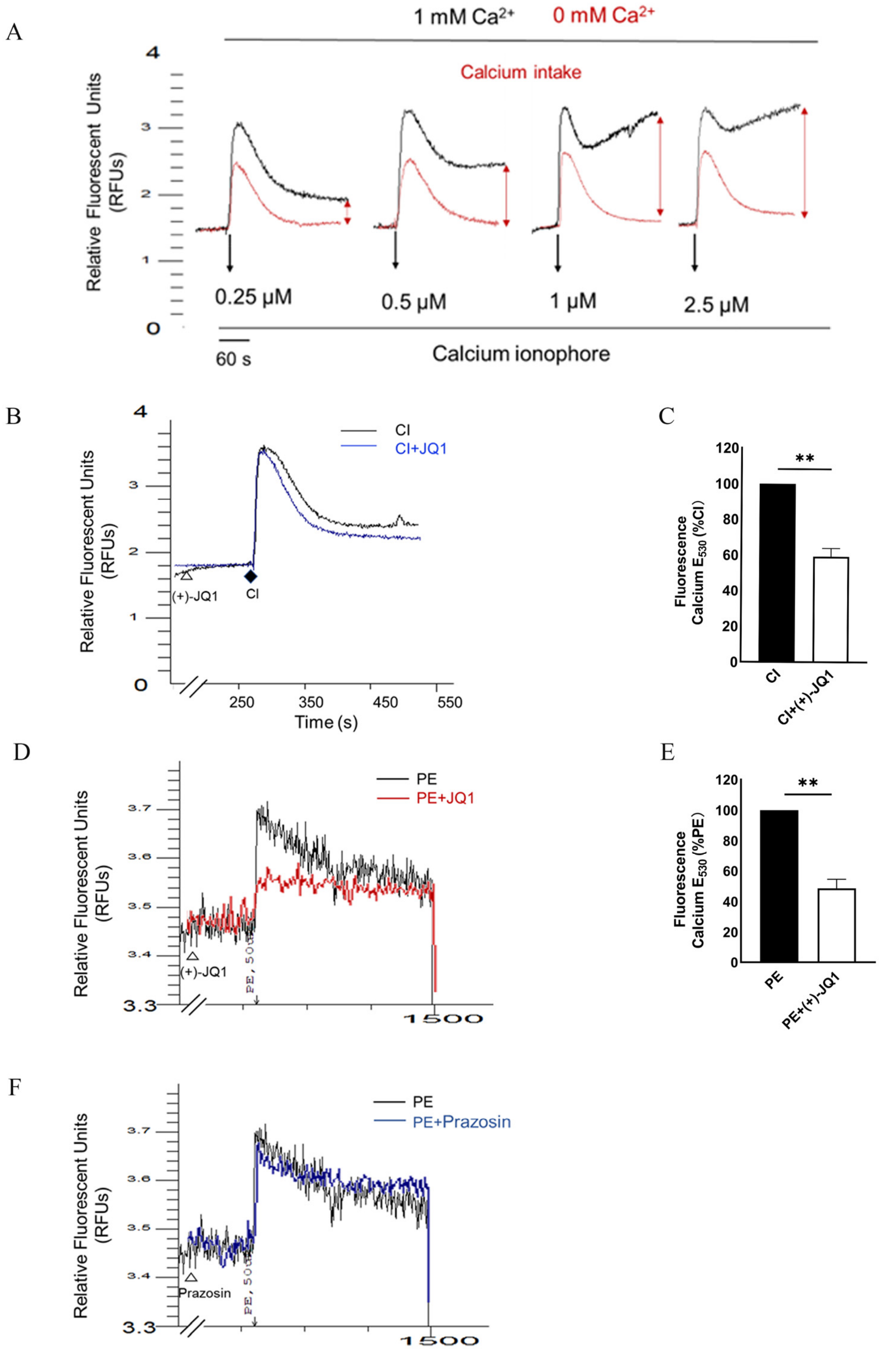

Supplemental Figure S5. (+)-JQ1 suppresses the influx of extracellular Ca^{2+}.

A: Original tracing illustrating dose-dependent CI–triggered calcium signaling with 530nm fluorescence emission in mouse primary SMCs in the 1 mM Ca^{2+} buffer (black tracing) or in Ca^{2+}-free buffer (red tracing). The red double arrow indicated the relative extracellular calcium intake in mouse primary SMCs stimulated by indicated concentrations of CI. **B**: Mouse primary SMCs were pretreated with (+)-JQ1 (50 μM) or DMSO for 5 min, then challenged with 0.5 μM CI in 1 mM Ca^{2+} buffer. **C**: Cumulative data showing the inhibition of (+)-JQ1 on the influx of extracellular Ca^{2+} evoked by CI. **$P<0.01$, n= 3. **D:** Mouse primary SMCs were pretreated with (+)-JQ1 (50 μM) or DMSO for 5 min, followed by adding

PE (50 μM) in 1 mM Ca^{2+} buffer. **E:** Cumulative data showing the inhibition of (+)-JQ1 on the PE-induced influx of extracellular Ca^{2+}. **P<0.01, n= 3. **F:** Mouse primary SMCs were pretreated with prazosin (5 μM) or vehicle for 5 min, then challenged by PE (50 μM) in 1mM Ca^{2+} buffer.

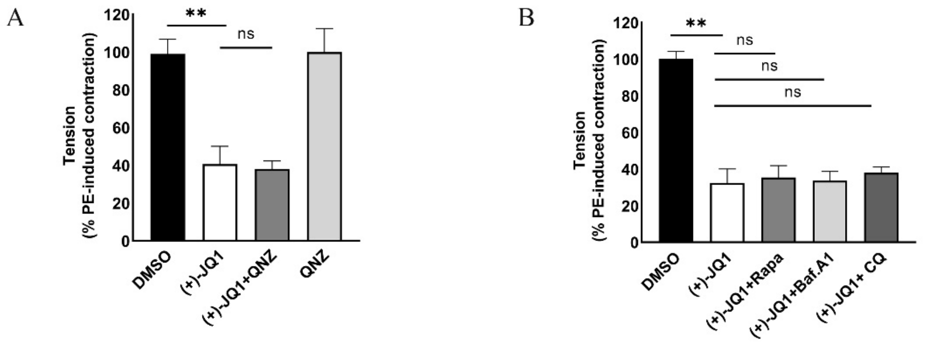

Supplemental Figure S6. (+)-JQ1 attenuates PE-induced contractile responses independent of the NF-κB pathway and autophagy. Mouse aortic rings were treated with (+)-JQ1 (10 μM) for 20 min after the application of QNZ (NF-κB inhibitor, 10 μM, A), rapamycin (autophagy activator, 100 nM, B), Baf.A1 (autophagy inhibitors,100 nM, B) and CQ (autophagy inhibitors, 30 μM, B), respectively, pretreatment for 20 min as indicated, followed by exposure to PE (1 μM). **P$<$0.01; ns: not significant; n=3.

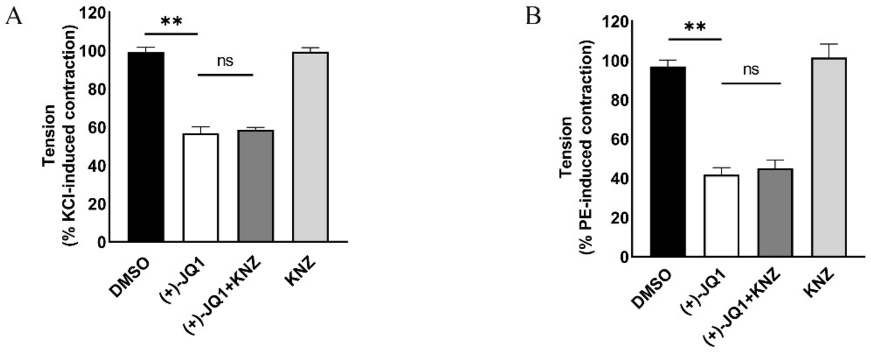

Supplemental Figure S7. Ketoconazole does not affect (+)- JQ1-induced inhibitory effects on KCl and PE-induced contractility. Mouse aortic tissues with intact endothelium were pretreated without and with KNZ (1 μM) for 20 min, followed by incubation (+)- JQ1 (10 μM) and subsequent exposure to KCl (A) or PE (B). ** p < 0.01, ns: not significant; n= 3.

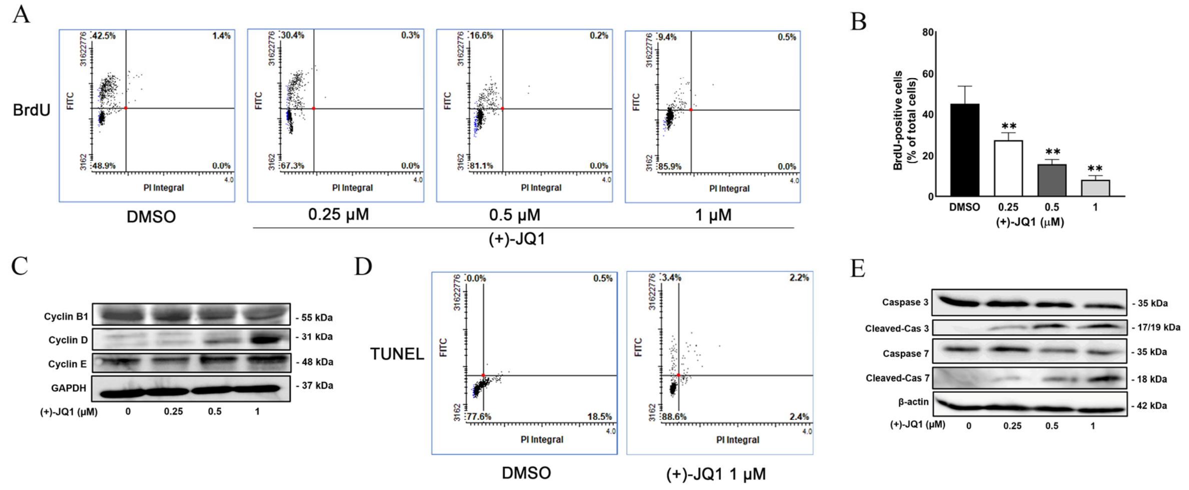

Supplemental Figure S8: (+)-JQ1 inhibits the cell cycle and promotes apoptosis in rat aortic SMCs. A: Primarily cultured rat aortic SMCs were grown on coverslips and exposed to various concentrations of (+)-JQ1 for a period of 24 hours. Afterward, they were pulse-labeled with BrdU (10 μM) for 60 minutes. This was followed by immunostaining with a BrdU monoclonal antibody and an Alexa Fluor 488-conjugated secondary antibody. The nuclei were then counterstained with PI. BrdU incorporation in each cell was subsequently analyzed with a Laser Scanning Cytometer (LSC, CompuCyte Corp, Cambridge, MA), as we previously described (PMID: 36750553). In the representative LSC scattergrams, the y-axis denotes the fluorescence intensity for incorporated BrdU, and the x-axis represents the total DNA content in each cell. BrdU-positive cells in the upper two quadrants are calculated as a percentage of the total cells. **B**: The LSC data cumulatively displays the percentage of BrdU-positive cells following (+)-JQ1 treatment. ** $p < 0.01$, n= 3. **C**: Primarily cultured rat aortic SMCs were treated with various concentrations of (+)-JQ1 for 24 hours, followed by WB detection of Cyclin B1, Cyclin D, Cyclin E, and β-actin. **D**: Representative LSC scattergrams for apoptotic cells are shown. Apoptosis of primarily cultured rat aortic SMCs treated with (+)-JQ1 (1 μM) for 24 hours was assessed using TUNEL assays, and this was followed by LSC analysis. In the LSC scattergrams, the y-axis represents the fluorescence intensity of TUNEL positive cells, while the x-axis is the total DNA content in each cell. **E**: Primarily cultured rat aortic SMCs were treated with different concentrations of (+)-JQ1 for 24 hours, followed by WB detection of cleaved caspase 3, caspase 3, cleaved caspase 7, caspase, and β-actin.
